# Association Between Complications and Death Within 30 Days After Orthopedic Surgery: Vascular Events in Noncardiac Surgery Patients Cohort Evaluation (VISION) Substudy

**DOI:** 10.2196/90823

**Published:** 2026-06-09

**Authors:** Lily J Park, P J Devereaux, Ameen Patel, Vikas Tandon, Diane Heels-Ansdell, Lehana Thabane, Pablo E Serrano, Matthew T V Chan, Wojciech Szczeklik, Sadeesh Srinathan, Ignacio Garutti, Gerard Urrutia, Ernesto Guerra-Farfan, Hassaan Abdel Khalik, Emmanuelle Duceppe, Sandra Ofori, Maura Marcucci, David Conen, Michael K Wang, Jessica Spence, Daniel Tushinski, Kamal Bali, Anthony Adili, Vickas Khanna, Ana Claudia Tonelli, Francesca Mulazzani, Wenjun Jiang, Olufemi R Ayeni, Gerard Slobegean, Theodore Miclau, Mohit Bhandari, Flavia K Borges

**Affiliations:** 1 Department of Surgery, Division of General Surgery, McMaster University, Hamilton, ON, Canada; 2 Population Health Research Institute, 237 Barton St E, C1-109, Hamilton, ON, Canada, 1 (905) 527-4322 ext 40654; 3 Division of Cardiology, Department of Medicine, McMaster University, Hamilton, ON, Canada; 4 World Health Research, Hamilton, ON, Canada; 5 Department of Medicine, McMaster University, Hamilton, ON, Canada; 6 Department of Health Research Methods, Evidence & Impact, McMaster University, Hamilton, ON, Canada; 7 Chinese University of Hong Kong, Hong Kong, China (Hong Kong); 8 Centre for Intensive Care and Perioperative Medicine, Jagiellonian College, Krakow, Kujawsko-Pomorskie, Poland; 9 Department of Surgery, University of Manitoba, Winnipeg, MB, Canada; 10 Department of Medicine, Universidad Complutense de Madrid, Madrid, Spain; 11 Institut de Recerca Sant Pau (IR SANT PAU) – CIBERESP, Barcelona, Spain; 12 Department of Surgery, Division of Orthopedic surgery, Vall d'Hebron Hospital Universitari, Barcelona, Catalonia, Spain; 13 Department of Surgery, Division of Orthopedic Surgery, McMaster University, Hamilton, ON, Canada; 14 Department of Medicine, Université de Montréal, Montreal, QC, Canada; 15 Department of Anesthesia, McMaster University, Hamilton, ON, Canada; 16 Internal Medicine Service, Hospital de Clínicas de Porto Alegre, Porto Alegre, Rio Grande do Sul, Brazil; 17 Department of Medicine, University of Milano-Bicocca, Milan, Lombardy, Italy; 18 Faculty of Health Sciences, McMaster University, Hamilton, ON, Canada; 19 Department of Orthopedics, School of Medicine, University of California, Irvine, CA, United States; 20 Department of Orthopaedic Surgery, University of California, San Francisco, San Francisco, CA, United States

**Keywords:** perioperative medicine, orthopedic surgery, morbidity, postoperative complications, mortality

## Abstract

**Background:**

The contemporary causes of postoperative mortality in orthopedic surgery are not well characterized.

**Objective:**

This study aimed to describe the epidemiology of postoperative complications among adult patients who underwent orthopedic surgery and inform their relationships with 30-day mortality.

**Methods:**

Vascular Events in Noncardiac Surgery Patients Cohort Evaluation (VISION) was a prospective cohort study involving 40,004 adult patients who underwent noncardiac surgery across 28 centers in 14 countries. For the subset of patients who underwent orthopedic surgery, a Cox proportional hazards model was used to determine time-dependent associations between various surgical complications and 30-day postoperative mortality. Analyses were adjusted for preoperative and surgical variables.

**Results:**

Among 8385 patients who underwent an orthopedic surgery in VISION, 1.6% (n=132) died within 30 days of surgery. Of the 132 deaths, 63.6% (n=84) occurred in hospital during the index hospitalization, while 36.4% (n=48) occurred after discharge. The incidence of death across the subcategories of orthopedic surgery was above-knee amputation (30/221, 13.6%), internal fixation of femur (29/750, 3.9%), lower leg amputation (9/252, 3.6%), major hip or pelvic surgery (49/2898, 1.7%), major spine surgery (8/1405, 0.6%), and knee arthroplasty (7/2876, 0.2%). A total of 6 postoperative complications (myocardial injury after noncardiac surgery [MINS], major bleeding, infection without sepsis, sepsis, stroke, and atrial fibrillation) were associated with death in adjusted analyses. The greatest attributable fractions of postoperative mortality (ie, proportion of deaths in the cohort that can be attributed to each complication, if causality were established) were from MINS (1454/8385, 17.3%; hazard ratio [HR] 2.08, 95% CI 1.38‐3.14; *P*<.001; attributable fraction=20.6%), major bleeding (2422/8385, 28.9%; HR 1.95, 95% CI 1.34‐2.85; *P*<.001; attributable fraction=16.5%), and sepsis (318/8385, 3.8%; HR 6.24, 95% CI 3.85‐10.12; *P*<.001; attributable fraction=9.7%).

**Conclusions:**

The complications most attributable to 30-day mortality following orthopedic surgery were MINS, major bleeding, and sepsis. These findings highlight areas for further study to mitigate perioperative mortality in orthopedic surgery. MINS demonstrated the highest attributable fraction for mortality (20.6%), emphasizing the importance of appropriate MINS screening, diagnosis, and management.

## Introduction

Globally, the number of orthopedic surgeries performed annually is estimated to have approached 31.4 million procedures in 2024 [1]. Among these patients, the mortality rate is broadly estimated to be between 0.6% to 8.7%. Even at the lower end of this estimated range, mortality after orthopedic surgery represents a substantial health issue [2-5]. Much of the existing mortality data are derived from patients with hip fracture and orthopedic trauma, which represents only a small fraction of the entire orthopedic surgery population [2-5]. This highlights the need to better elucidate mortality rates in a broad sample of general contemporary patients who underwent orthopedic surgery.

The largest study to date in this area used the National Hospital Discharge Survey to identify risk factors for mortality in orthopedic surgery across a nationwide sample of hospitals in the United States [5]. However, this study is limited by the retrospective nature of the study, reliance on administrative data, and older data. Furthermore, the National Hospital Discharge Survey is considered less reliable in reflecting morbidity and complications [5-10]. Considering this, an updated and accurate understanding of modifiable risk factors for death in orthopedic surgery is needed.

Vascular Events in Noncardiac Surgery Patients Cohort Evaluation (VISION) was a large prospective cohort study that included patients who underwent noncardiac surgery and were systematically followed to document postoperative complications, including mortality [11]. We previously reported the incidence of perioperative complications and associated mortality for the entire VISION study population. Patients undergoing orthopedic surgery represent a unique population that increasingly includes older adults with rising prevalence of cardiovascular disease [12-14]. This necessitates specialty-specific epidemiologic data.

The objective of this prospective cohort substudy was to describe the epidemiology of postoperative complications and death, evaluate the associations between these postoperative complications and death, and report the attributable fractions of each complication for death among a contemporary orthopedic surgery cohort.

## Methods

### Ethical Considerations

The research ethics board at each participating site approved the protocol before patient recruitment. This study was approved by the Hamilton Integrated Research Ethics Board under the project number 07-220. All participants provided informed consent and we followed the ethical standard principles of the Declaration of Helsinki, including maintaining privacy and confidentiality of research participants' data. No compensation was provided to participants.

### Study Design

The design and methods of the VISION study have been previously described [11,15]. In summary, VISION was an international prospective cohort study, which enrolled 40,004 patients who underwent noncardiac surgery across 28 centers in 14 countries from August 2007 to November 2013. Patients aged ≥45 years, who underwent noncardiac surgery, receiving general or regional anesthesia, and requiring at least 1 overnight hospital stay after surgery were eligible for inclusion. This inclusion criteria was determined a priori with the intention to efficiently identify those who are at higher risk of mortality and capture a greater number of mortality outcomes and perioperative complications. Each participating hospital obtained approval from their research ethics board prior to the start of patient enrollment. Refer to Multimedia Appendix 1 for funding sources. The Strengthening the Reporting of Observational Studies in Epidemiology Statement was followed (Checklist 1).

### Analysis Population

Patients who were enrolled in the VISION study and underwent an orthopedic procedure (including spine surgery) were included in this substudy. According to the VISION definitions, this would include surgeries categorized as major hip or pelvic surgery, internal fixation of femur, knee arthroplasty, above-knee amputation, lower leg amputation, and major spine surgery.

### Follow-Up

Patients were followed for 30 days following their surgery and were censored at the time of their last assessment if the 30-day follow-up was not complete.

### Complications Variables

Definitions for all variables included in the analyses are provided in (Multimedia Appendix 2). The primary outcome was time to all-cause mortality. The following postoperative complications during the first 30 days after surgery were investigated: myocardial injury after noncardiac surgery (MINS), venous thromboembolism (VTE), stroke, major bleeding, acute kidney injury (AKI) resulting in dialysis, sepsis, infection without sepsis, new clinically important atrial fibrillation, and congestive heart failure [11,12,16-19]. These variables were selected for clinical relevance while being parsimonious to preserve model stability. Of note, major bleeding was defined using a previously validated definition in the perioperative setting demonstrating independent association with 30-day mortality [18,19].

### Statistical Analysis

A statistical analysis plan was written before undertaking analyses for this study, which was finalized in June 2023. Descriptive statistics were used to report patient characteristics, as well as the incidence of death and postoperative complications. As AKI resulting in dialysis occurred at the rate of 0.2%, this variable was omitted from the model to preserve model stability.

To determine the relationship between complications and 30-day mortality, we used a time-dependent Cox proportional hazards model and adjusted for baseline characteristics that were known to be independently associated with 30-day mortality, according to previous VISION analyses [11]. Complications were modeled as time-dependent covariates, allowing for multiple events per patient and accounting for their timing relative to death. This approach enabled assessment of the association between each complication with mortality rather than assignment of a single causal event. This model was adjusted for age category (65‐75 vs 45‐65 years, and age ≥75 vs 45‐65 years), cancer at the time of surgery, history of chronic obstructive pulmonary disease, surgery urgency (emergent: <24 hours from admission for an acute surgical condition, urgent: 24‐72 hours from admission for an acute surgical condition, nonemergent: all other surgeries), and history of peripheral arterial disease. All variables included in the model were selected a priori. We reported the adjusted hazard ratios (HRs) and corresponding 95% CIs. To avoid optimistic estimation of the c-statistic from possible overfitting, model performance was assessed using the c-statistic corrected for optimism using 1000 bootstrapped samples [20]. This involved the generation of 1000 simulated datasets through random sampling and replacement from the original dataset to estimate model performance while accounting for overfitting. Attributable fractions for death were calculated using an established method [21]. The attributable fraction represents the proportion of deaths that would not have occurred in the VISION orthopedic cohort if the complication had not occurred, if we assumed a causal relationship between the complication and death. Attributable fraction methods have been increasingly used in perioperative and epidemiologic research. Prior applications include large cohort analyses in noncardiac surgery and population health studies evaluating modifiable contributors to mortality [11,17,21-23]. There were 10 events per variable included in the model, which supports model stability [24].

For all tests, we used α <.05 as the level of significance. Analyses were performed using R statistical software (version 4.2.2; R Foundation for Statistical Computing) using the “survival,” “epiR,” and “ggplot2” packages.

## Results

### Patient Characteristics

**Figure 1.**
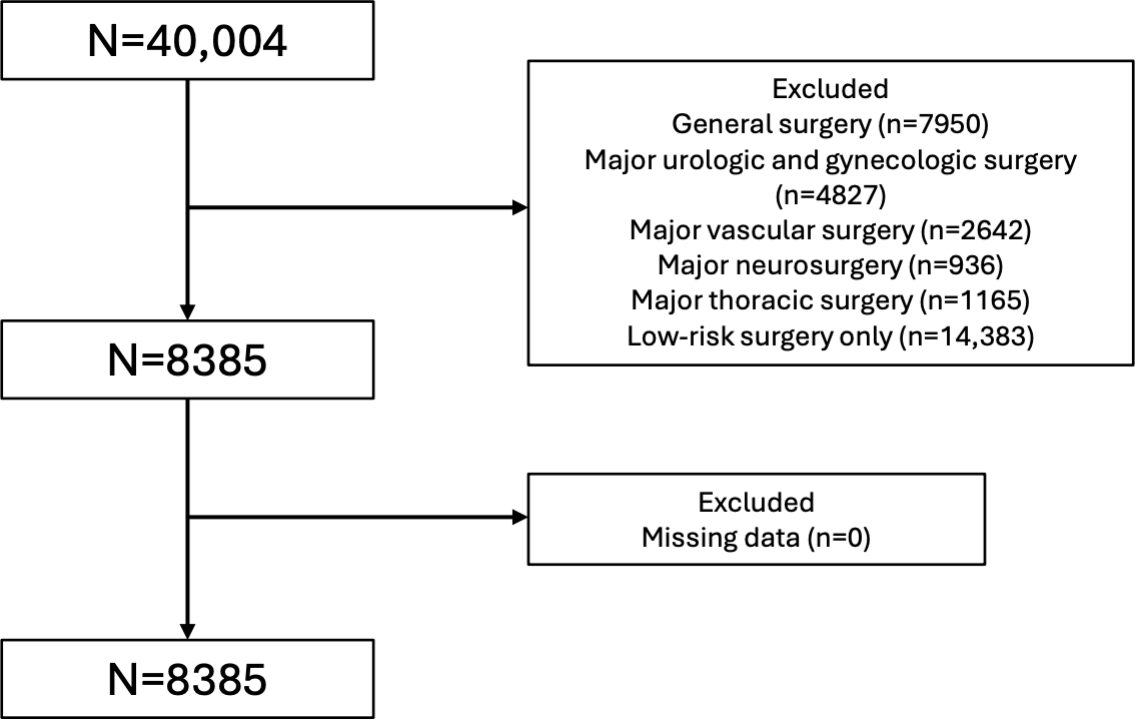
Flow of patient inclusion in the Vascular Events in Noncardiac Surgery Patients Cohort Evaluation (VISION) orthopedic surgery substudy. Note: in the overall VISION study, 284 patients had 2 or 3 surgical categories.

Within the 40,004 patients enrolled in the overall VISION study, 21% (8385/40,004) underwent an orthopedic surgery and were included in these analyses. Figure 1 demonstrates the flow of patient inclusion. Table 1 presents the baseline characteristics of the participants. More than half of the patients were aged >64 years, and 57.3% (4802/8385) were female. There was no loss to follow-up in the orthopedic surgery population.

**Table 1.** Baseline characteristics of the orthopedic surgery cohort.

Characteristics	Participants, n (%)	Number of deaths within 30 days, n (%)
Age (years)		
45‐64	3411 (40.7)	31 (0.9)
65‐74	2418 (28.8)	20 (0.8)
≥75	2556 (30.5)	81 (3.2)
Sex: female	4802 (57.3)	76 (1.6)
History of		
Hypertension	4948 (59)	89 (1.8)
Diabetes	1823 (21.8)	44 (2.4)
Coronary artery disease	1253 (15)	41 (3.3)
Peripheral arterial disease	519 (6.2)	39 (7.5)
Chronic obstructive pulmonary disease	640 (7.6)	33 (5.2)
Coronary revascularization	470 (5.6)	6 (1.3)
Stroke	398 (4.7)	20 (5)
Congestive heart failure	401 (4.8)	20 (5)
Active cancer	320 (3.8)	13 (4.1)
Atrial fibrillation	323 (3.9)	11 (3.4)
Preoperative estimated glomerular filtration rate (mL/min/1.73 m^2^)		
<30	294 (3.7)	26 (8.8)
30‐44	503 (6.2)	22 (4.4)
45‐59	988 (12.3)	12 (1.2)
≥60	6264 (77.8)	67 (1.1)
Types of orthopedic surgery		
Major hip or pelvic surgery	2898 (34.6)	49 (1.7)
Internal fixation of femur	750 (8.9)	29 (3.9)
Knee arthroplasty	2876 (34.3)	7 (0.2)
Above-knee amputation	221 (2.6)	30 (13.6)
Lower leg amputation	252 (3)	9 (3.6)
Major spine surgery	1405 (16.8)	8 (0.6)
Subcategory of surgery		
Nonemergent	7369 (87.9)	81 (1.1)
Urgent or emergent	1016 (12.1)	51 (5)
Type of anesthesia		
General only	2436 (29.1)	36 (1.5)
Neuraxial (spinal or epidural) only	4182 (49.9)	72 (1.7)
General with nitrous oxide only	424 (5.1)	10 (2.4)
General and thoracic epidural only	10 (0.1)	0 (0)
General and nerve block only	424 (5.1)	2 (0.5)
Other	903 (10.8)	12 (1.3)

Among 8385 patients, 34.6% (n=2898) underwent a major hip or pelvic surgery, 34.3% (n=2876) underwent knee arthroplasty, 16.8% (n=1405) underwent a major spine surgery, 8.9% (n=750) underwent internal fixation of femur, 3% (n=252) underwent lower leg amputation, and 2.6% (n=221) underwent above-knee amputation. The majority of patients (n=7370, 87.9%) underwent nonemergent surgery.

### Incidence of Death Within 30 Days After Surgery

There were 132 (1.6%) deaths in the orthopedic surgery cohort within 30 days after surgery (Table 2). Of the 132 deaths, 63.6% (84/132) occurred in hospital during the index hospitalization, while the remaining 36.4% (48/132) occurred after discharge. In 1.5% (2/132) of the deaths, patients died in the operating room. The median time to death was 13.5 (IQR 6‐21) days. Refer to Table 2 for incidence of death according to subcategories of orthopedic surgery. Death rates by nonemergent, urgent, and emergent surgeries were 1.1% (81/7288), 5.7% (46/812), and 3.3% (5/153), respectively. Risk of 30-day mortality was increased in emergent and urgent surgeries compared to nonemergent surgeries in adjusted analyses (HR 2.30, 95% CI 1.61‐3.42; *P*<.001).

**Table 2.** Thirty-day perioperative complications, overall and by subtypes of orthopedic surgery^a^.

Outcome	Patients, n (%)						
	All orthopedic surgery (N=8385)	Subtypes of orthopedic surgery					
		Major hip or pelvic surgery (n=2898)	Internal fixation of femur (n=750)	Knee arthroplasty (n=2876)	Above-knee amputation (n=221)	Lower leg amputation (n=252)	Major spine surgery (n=1405)
Major bleeding	2422 (28.9)	965 (33.3)	287 (38.3)	725 (25.2)	103 (46.6)	96 (38.1)	258 (18.4)
MINS^b^	1454 (17.3)	563 (19.4)	165 (22)	347 (12.1)	95 (43)	95 (37.7)	197 (14)
Sepsis	318 (3.8)	124 (4.3)	33 (4.4)	53 (1.8)	30 (13.6)	20 (7.9)	60 (4.3)
Infection without sepsis	562 (6.7)	251 (8.7)	63 (8.4)	153 (5.3)	14 (6.3)	29 (11.5)	54 (3.8)
Acute kidney injury with dialysis	17 (0.2)	4 (0.1)	3 (0.4)	2 (0.1)	2 (0.9)	3 (1.2)	3 (0.2)
Stroke	27 (0.3)	9 (0.3)	6 (0.8)	6 (0.2)	1 (0.5)	2 (0.8)	3 (0.2)
Venous thromboembolism	123 (1.5)	29 (1)	16 (2.1)	69 (2.4)	1 (0.5)	0 (0)	8 (0.6)
Congestive heart failure	124 (1.5)	50 (1.7)	29 (3.9)	23 (0.8)	13 (5.9)	6 (2.4)	4 (0.3)
New, clinically important atrial fibrillation	93 (1.1)	46 (1.6)	13 (1.7)	28 (1)	2 (0.9)	1 (0.4)	4 (0.3)
Death	132 (1.6)	49 (1.7)	29 (3.9)	7 (0.2)	30 (13.6)	9 (3.6)	8 (0.6)

a Subtypes of orthopedic surgery were not mutually exclusive (ie, 1 patient could have been categorized to have undergone more than 1 subtype of orthopedic surgery).

b MINS: myocardial injury after noncardiac surgery.

### Postoperative Complications and Relationship with 30-Day Mortality

The most common complication was major bleeding (2422/8385, 28.9% patients), followed by MINS (1454/8385, 17.3% patients), infection without sepsis (562/8385, 6.7% patients), sepsis (318/8385, 3.8% patients), then VTE (123/8385, 1.5% patients), congestive heart failure (124/8385, 1.5% patients), new atrial fibrillation (93/8385, 1.1% patients), stroke (27/8385, 0.3% patients), and AKI resulting in dialysis (17/8385, 0.2% patients). Refer to Table 2 for details. The median time from surgery to major bleeding was 2 (IQR 1‐3) days, MINS 2 (IQR 1‐3) days, infection without sepsis 10 (IQR 5‐17) days, and sepsis 8 (IQR 4‐13) days. Tables3  4 demonstrate the proportion of patients that underwent surgery for an acute fracture as well as the mortality rates according to the timing of surgery performed within each subcategory of orthopedic surgery.

**Table 3.** Surgery for acute fracture according to subcategories of orthopedic surgery^a^.

Subcategory of orthopedic surgery	Surgery for acute fracture^b^, n (%)	
	No (n=6767)	Yes (n=1616)
Major hip or pelvic surgery^c^	1982 (29.3)	916 (56.7)
Internal femoral fixation	119 (1.8)	622 (38.5)
Knee arthroplasty	2854 (42.2)	21 (1.3)
Above-knee amputation	216 (3.2)	3 (0.2)
Lower leg amputation	243 (3.6)	6 (0.4)
Major spine surgery	1353 (20)	48 (3)

a Surgery for acute fracture was considered nonelective surgery.

b 2 patients with missing subcategory of orthopedic surgery.

c Surgery for hip and pelvis were not mutually exclusive.

**Table 4.** Mortality rates according to timing of surgery (emergent or urgent vs nonemergent surgery) within each subcategory of orthopedic surgery.

Subcategory and timing of surgery	Proportions^a^, n (%)^b^	Mortality, n	Mortality rate^c^, % (95% CI)
Major hip or pelvic surgery (N=2898)			
Emergent or urgent	525 (18.1)	22	4.2 (2.7‐6.4)
Nonemergent	2373 (81.9)	27	1.13 (0.8‐1.7)
Internal femoral fixation (N=741)			
Emergent or urgent	313 (42.2)	14	4.5 (2.6‐7.6)
Nonemergent	428 (57.8)	15	3.5 (2.1‐5.8)
Knee arthroplasty (N=2875)			
Emergent or urgent	35 (1.2)	0	0
Nonemergent	2840 (98.8)	7	0.24 (0.1‐0.5)
Above-knee amputation (N=219)			
Emergent or urgent	48 (21.9)	11	23 (12.5‐37.7)
Nonemergent	171 (78.1)	19	11.2 (7.0‐17.0)
Lower leg amputation (N=249)			
Emergent or urgent	49 (19.7)	3	6 (1.6‐17.9)
Nonemergent	200 (80.3)	6	3.0 (1.2‐6.7)
Major spine surgery (N=1403)			
Emergent or urgent	46 (3.3)	1	2 (0.1‐13.0)
Nonemergent	1357 (96.7)	7	0.51 (0.2‐1.1)
All (N=8385)			
Emergent or urgent	1016 (12.1)	51	5.01 (3.8‐6.6)
Nonemergent	7369 (87.9)	81	1.09 (0.9‐1.4)

a 2 patients with missing subcategory of orthopedic surgery

b The proportion was calculated using the total number of patients within each surgical subcategory as the denominator.

c The proportion is out of total patients receiving urgent or emergent surgery versus nonemergent, within each subcategory of surgery.

**Figure 2.**
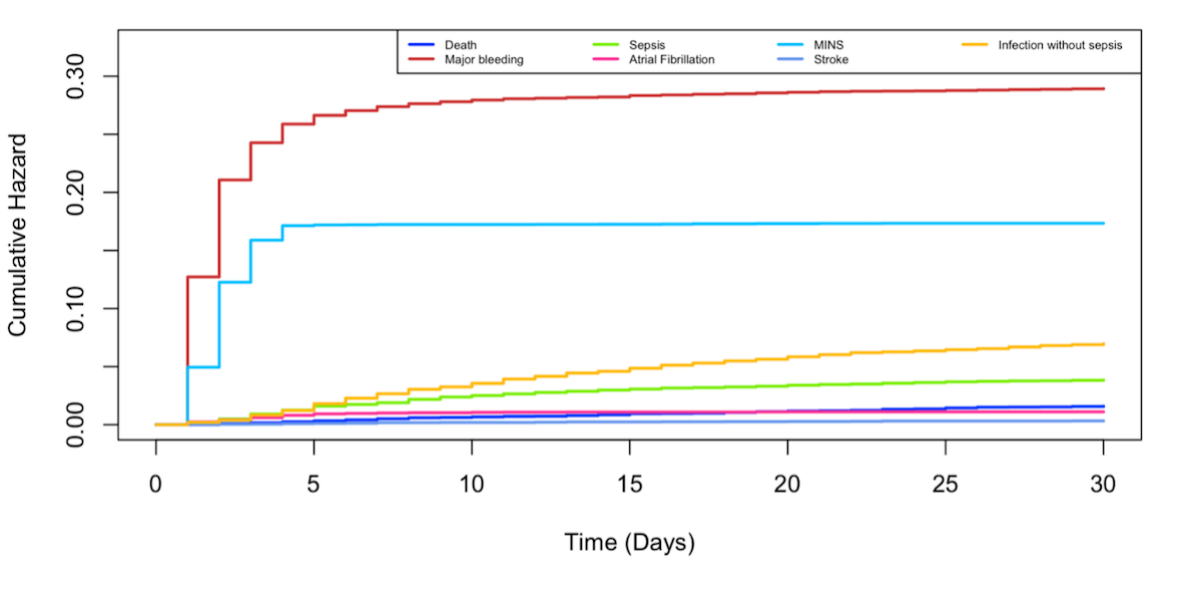
Cumulative hazard curve of postoperative complications associated with 30-day mortality in orthopedic surgery. MINS: myocardial injury after noncardiac surgery.

Postoperative complications associated with 30-day mortality included major bleeding (adjusted hazard ratio [aHR] 1.95, 95% CI 1.34‐2.85), MINS (aHR 2.08, 95% CI 1.38‐3.14), sepsis (aHR 6.24, 95% CI 3.85‐10.12), infection without sepsis (aHR 2.74, 95% CI 1.54‐4.85), stroke (aHR 6.01, 95% CI 2.19‐16.56), and new clinically important atrial fibrillation (aHR 2.65, 95% CI 1.25‐5.65). Refer to Table 5 for details. The c-statistic for model performance before and after correction for optimism was 0.87 and 0.85, respectively. Figure 2 is a cumulative hazard curve of the postoperative complications associated with 30-day mortality.

**Table 5.** Relation between perioperative complications and 30-day mortality in orthopedic surgery.

Outcome	Patients who died, % (n) (95% CI)	Adjusted HR^a^^b^ (95% CI)	Attributable fraction (%)
Major bleeding (n=2422)	3.1 (75) (2.44‐3.87)	1.95 (1.34‐2.85)	16.5
No major bleeding (n=5960)	0.9 (57) (0.73‐1.24)	Reference	N/A^c^
MINS^d^ (n=1454)	4.3 (63) (3.35‐5.51)	2.08 (1.38‐3.14)	20.6
No MINS (n=6931)	0.9 (69) (0.78‐1.26)	Reference	N/A
Sepsis (n=318)	9.4 (30) (6.46‐13.19)	6.24 (3.85‐10.12)	9.7
Infection without sepsis (n=562)	3 (17) (1.77‐4.80)	2.74 (1.54‐4.85)	3.8
No sepsis or infection (n=7503)	1.1 (85) (0.91‐1.40)	Reference	N/A
Acute kidney injury with dialysis (n=17)	41.2 (7) (18.44‐67.07)	N/A	N/A
No acute kidney injury with dialysis (n=8366)	1.5 (125) (1.25‐1.78)	N/A	N/A
Stroke (n=27)	14.8 (4) (4.19‐33.73)	6.01 (2.19‐16.56)	1.5
No stroke (n= 8356)	1.5 (128) (1.28‐1.82)	Reference	N/A
Venous thromboembolism (n=123)	2.4 (3) (0.51‐6.96)	2.24 (0.70‐7.13)	N/A
No venous thromboembolism (n=8261)	1.6 (129) (1.31‐1.85)	Reference	N/A
Congestive heart failure (n=124)	15.3 (19) (9.48‐22.89)	1.54 (0.81‐2.94)	N/A
No congestive heart failure (n=8259)	1.4 (13) (1.13‐1.64)	Reference	N/A
New, clinically important atrial fibrillation (n=93)	10.8 (10) (5.28‐18.89)	2.65 (1.25‐5.65)	2.2
No new, clinically important atrial fibrillation (n=8290)	1.5 (122) (1.22‐1.75)	Reference	N/A

a HR: hazard ratio.

b Adjusted variables were as follows: age, history of peripheral vascular disease, history of chronic obstructive pulmonary disease, surgery urgency, and active cancer.

c N/A: not applicable.

d MINS: myocardial injury after noncardiac surgery.

Among the postoperative complications significantly associated with mortality, the greatest attributable fraction was for MINS (20.6%), followed by major bleeding (16.5%), then sepsis (9.7%), infection without sepsis (3.8%), new atrial fibrillation (2.2%), and stroke (1.5%).

Post hoc subgroup analyses performed according to surgery urgency demonstrated similar findings. Among those who underwent nonemergent surgeries, there were 81 deaths. Following adjustment, MINS (HR 2.02, 95% CI 1.22‐3.32), atrial fibrillation (HR 2.64, 95% CI 1.04‐6.89), major bleeding (HR 2.30, 95% CI 1.41‐3.76), sepsis (HR 6.27, 95% CI 3.43‐11.46), and stroke (HR 10.49, 95% CI 3.18‐34.63) were significantly associated with 30-day mortality. Among those who underwent urgent and emergent surgeries, there were 51 deaths. In this subgroup, with further reduced number of mortality events, infection without sepsis (HR 3.49, 95% CI 1.85‐6.24), MINS (HR 3.40, 95% CI 1.85‐6.24), and sepsis (HR 5.65, 95% CI 2.51‐12.70) were significantly associated with 30-day mortality.

## Discussion

### Principal Findings

This large international prospective cohort study involving 8385 patients who underwent orthopedic surgery demonstrated a 1.6% (132/8385) mortality rate. Major bleeding, MINS, infection without sepsis, sepsis, stroke, and new clinically important atrial fibrillation were significantly associated with 30-day mortality. Among these, the greatest attributable fraction for death in our cohort was from MINS (1454/8385, 21%), major bleeding (2422/8385, 17%), sepsis (318/8385, 10%), infection without sepsis (562/8385, 4%), stroke (27/8385, 2%), and new atrial fibrillation (93/8385, 2%).

Consistent with prior studies that reported 30-day mortality rates ranging from 0.6% to 8.7%, this work demonstrated a 1.6% (132/8385) mortality rate [2-4]. Our study also demonstrated substantial variations in mortality across subcategories of orthopedic surgery and surgery urgency. Traditional preoperative risk scoring systems do not consider the nuances between surgical procedures that contribute to notable variations in mortality risk [25]. We found substantial mortality differences across surgical procedures within orthopedic surgery, which urges greater consideration of procedure type for preoperative risk stratification and consideration of higher-level postoperative monitoring (eg, admission to intensive cardiac care unit and telemetry).

Among the few studies that investigate complications associated with mortality in orthopedic surgery, pneumonia, acute renal failure, stroke, myocardial infarction, and MINS have been reported [5,26]. Our results are unique in identifying major bleeding to be a risk factor for death in the orthopedic surgery cohort. Previous studies did not select postoperative bleeding as a potential variable to explore, despite 80% of 739 orthopedic surgeons and 50 anesthesiologists in an international survey responding that they were either concerned or very concerned for bleeding in orthopedic surgery populations [27]. Our results demonstrate that major bleeding in orthopedic surgery is common and is associated with mortality, necessitating further investigations in this area to improve patient outcomes.

Although a major bleeding event was the most common complication in our cohort, MINS demonstrated the greatest attributable fraction for death. In other words, although an overall smaller number of patients experienced MINS, a greater number of patients with MINS died within 30 days of surgery compared to the number of patients who experienced major bleeding. This is unique to the orthopedic surgery cohort compared to the overall VISION mortality analyses and general surgery mortality substudy, where major bleeding had the highest attributable fraction for death [11,17]. Certainly, the varying risk profiles, propensity for bleeding, and cardiovascular stress by surgical subspecialties contribute to the differences in these results. This emphasizes the need for specialty-specific data to inform surgery-specific practice.

Another potential explanation is the unique practice of bleeding prophylaxis that is commonplace in orthopedic surgery [28-31]. Consistent demonstration of perioperative bleeding reduction with tranexamic acid use in orthopedic surgery–specific research has led to the routine use of prophylactic tranexamic acid in orthopedic surgery for many years, particularly in trauma, joint, and spine surgery [28-33]. This is not commonplace in other noncardiac surgical specialties, despite recent evidence to support its safety and efficacy in these contexts [28-30,34-37]. The long-standing practice of routine prophylaxis for perioperative bleeding in orthopedic surgery may have contributed to reduced bleeding severity and lower attributable fraction for death in this cohort compared to the overall and general surgery cohorts. If this were to be true, it is also worth noting that the rate of VTE in this orthopedic cohort was only around 1% (123/8385), which is similar to the incidence found in the noncardiac and general surgery cohorts [11].

Of the postoperative complications investigated, MINS was the second most common complication occurring in 17.3% (1454/8385) of the orthopedic surgery cohort. We also demonstrated a 12% (347/2876) incidence of MINS in the knee arthroplasty subcategory. Previously reported rates of myocardial infarction (MI) after knee arthroplasty have ranged between 0.3% and 2.2% [38,39]. Considering MINS encompasses a broader spectrum of myocardial injury than MI, the larger incidence of MINS was expected, especially as high-sensitivity troponin assays were used for monitoring. Among the few existing studies investigating the incidence of MINS, the results have varied significantly, likely due to limitations in very small sample sizes (ie, 1/82, 1.2% incidence vs 68/160, 42% incidence) [40,41]. Our study included 2876 patients who underwent knee arthroplasty, representing the largest cohort in which the epidemiology of MINS has been studied. As MINS is largely asymptomatic, these findings highlight the importance of identifying MINS for postoperative risk stratification and downstream management, as emerging evidence suggests targeted treatment may reduce future cardiovascular events [12,16,25,42].

Finally, subgroup analyses by surgery urgency demonstrated minor differences in postoperative complications that were found to be associated with mortality between the emergent and nonemergent groups. Specifically, major bleeding was not associated with mortality in the urgent-emergent subgroup. Clinical interpretation of these findings is limited as these are post hoc analyses with reduced events per variable and therefore, model instability. However, these findings suggest differences in mortality determinants highlighting tailored strategies to address perioperative risks according to surgery urgency.

### Limitations

There are a few limitations to consider. Only patients aged ≥45 years were included; thus, these findings may not apply to younger patients. Baseline variables (ie, age and estimated glomerular filtration rate) were modeled as a categorical variable to align with prior VISION analyses, although this approach may not fully capture potential nonlinear relationships. Additionally, as our inclusion criteria were targeted to include a higher risk surgical population, the present results would not apply for patients undergoing low-risk, same-day surgeries. The granularity of the data are limited to the subcategories of orthopedic surgery that were predefined before the start of the study [43-45]. Although orthopedic procedures are heterogeneous, combining them enabled sufficient power to evaluate associations between relatively infrequent complications and mortality. We report subtype-specific complication rates (Table 2), demonstrating that major bleeding, MINS, and sepsis were consistently among the most common complications across procedures. While procedure-specific attributable fractions were not feasible due to limited events, our findings identify broadly relevant targets for perioperative risk reduction. The stability of a Cox regression model is dependent on the events per variable. The base model meets the lower acceptable cutoff at 10 events per variable, suggesting adequate power for the primary analysis; there remains potential risk for overfitting [24,46]. However, the negligible change in the c-statistic after correcting for optimism suggests overfitting may not be a significant issue [20]. Variation in VTE prophylaxis practices, which were not captured in detail in this study, may contribute to differences in bleeding risk and represents a potential unmeasured confounder. Finally, complications such as infection and sepsis may occur later in the postoperative course, potentially reducing their relative contribution in time-dependent analyses within a 30-day follow-up period, particularly given their lower event frequency compared to earlier complications such as bleeding or MINS.

To our knowledge, this is the first large prospective study exploring the association of various postoperative complications and death in a global prospective orthopedic surgery study. The inclusion of diverse participants across 14 countries increases the generalizability of our results. Furthermore, with no loss to follow-up and the collection of time-dependent data, we were able to increase precision and reduce bias in fulfilling our objectives by creating a realistic model that accounts for the changes in risk factors for survival that occur over time.

### Conclusions

This large international prospective cohort study of patients undergoing orthopedic surgery (7369/8385, 88% nonemergent) demonstrated a 30-day mortality rate of 1.6% (132/8385). Adjusted analyses demonstrated major bleeding, MINS, sepsis, infection without sepsis, stroke, and atrial fibrillation to be associated with mortality. The highest attributable fraction of death in our cohort was contributed by MINS, major bleeding, sepsis, and infection without sepsis, which highlights areas for further study to reduce mortality among patients who underwent orthopedic surgery.

## Supplementary material

10.2196/90823 Multimedia Appendix 1 VISION funding sources.

10.2196/90823 Multimedia Appendix 2 VISION postoperative complications and baseline variable definitions.

10.2196/90823 Checklist 1 STROBE checklist.

## Abbreviations

aHR: adjusted hazard ratio
AKI: acute kidney injury
HR: hazard ratio
MI: myocardial infarction
MINS: myocardial injury after noncardiac surgery
VISION: Vascular Events in Noncardiac Surgery Patients Cohort Evaluation
VTE: venous thromboembolism
