# Supplementary material for: Association Between Complications and Death Within 30 Days After Orthopedic Surgery: Vascular Events in Noncardiac Surgery Patients Cohort Evaluation (VISION) Substudy
Source: JMIR Perioper Med. 2026 Jun 9;9:e90823. doi: 10.2196/90823 (PMC13249062; doi:10.2196/90823)
Supplement: Multimedia Appendix 1 [file periop-v9-e90823-s001.docx]

**Appendix 1:** VISION Funding Sources

**Canada**

1.Canadian Institutes of Health Research – 7 grants

2.Heart and Stroke Foundation of Ontario – 2 grants

3.Academic Health Science Centres Alternative Funding Plan Innovation Fund Grant Ontario

4.Population Health Research Institute Grant

5.CLARITY Research Group Grant

6.McMaster University, Department of Surgery, Surgical Associates Research Grant

7.Hamilton Health Science New Investigator Fund Grant

8.Hamilton Health Sciences Grant

9.Ontario Ministry of Resource and Innovation Grant

10.Stryker Canada

11.McMaster University, Department of Anesthesiology – 2 grants

12.Saint Joseph’s Healthcare, Department of Medicine – 2 grants

13.Father Sean O’Sullivan Research Centre – 2 grants

14.McMaster University, Department of Medicine – 2 grants

15.Roche Diagnostics Global Office – 5 grants

16.Hamilton Health Sciences Summer Studentships – 6 grants

17.McMaster University, Department of Clinical Epidemiology and Biostatistics Grant

18.McMaster University, Division of Cardiology Grant

19.Canadian Network and Centre for Trials Internationally Grant

20.Winnipeg Health Sciences Foundation Operating Grant

21.University of Manitoba, Department of Surgery Research Grant – 2 grants

22.Diagnostic Services of Manitoba Research Grant

23.Manitoba Medical Services Foundation Grant

24.Manitoba Health Research Council Grant

25.University of Manitoba, Faculty of Dentistry Operational Fund

26.University of Manitoba, Department of Anesthesia Grant

27.University Medical Group, Department of Surgery, University of Manitoba, start-up Fund

**Australia**

28.National Health and Medical Research Council Program Grant

**Brazil**

29.Projeto Hospitais de Excelência a Serviço do SUS (PROADI-SUS) grant from the BrazilianMinistry of Health in Partnership with Hcor (Cardiac Hospital Sao Paulo-SP)

30.National Council for Scientific and Technological Development (CNPq), grant from theBrazilian Ministry of Science and Technology

31.National Institute for Health Technology Assessment - IATS/ CNPq grant

32.FIPE grant, Hospital de Clinicas de Porto Alegre

**China**

33.Public Policy Research Fund (CUHK-4002-PPR-3), Research Grant Council, Hong KongSAR

34.General Research Fund (461412), Research Grant Council, Hong Kong SAR

35.Australian and New Zealand College of Anaesthetists Grant (13/008), Melbourne, Australia

**Colombia**

36.School of Nursing, Universidad Industrial de Santander

37.Grupo de Cardiología Preventiva, Universidad Autónoma de Bucaramanga

38.Fundación Cardioinfantil – Instituto de Cardiología

39.Alianza Diagnóstica S.A.

**France**

40.Université Pierre et Marie Curie, Département d’anesthésie Réanimation, Pitié-Salpêtrière,Assistance Publique- Hôpitaux de Paris Grant

**India**

41.St. John's Medical College and Research Institute Grant, Division of Clinical Research andTraining Grant

**Malaysia**

42.University of Malaya Research Grant (RG302-14AFR)

43.University of Malaya, Penyelidikan Jangka Pendek Grant (PJP)

**Poland**

44. Polish Ministry of Science and Higher Education (NN402083939) Grant

**South Africa**

45. University of KwaZulu-Natal Grant

**Spain**

46. Instituto de Salud Carlos III (PI0790246)

47. Fundació La Marató de TV3 (082330)

**United States**

48. American Heart Association Grant

49. Covidien Grant

**United Kingdom**

50. National Institute for Health Research (NIHR)
