# Supplementary material for: Association Between Complications and Death Within 30 Days After Orthopedic Surgery: Vascular Events in Noncardiac Surgery Patients Cohort Evaluation (VISION) Substudy
Source: JMIR Perioper Med. 2026 Jun 9;9:e90823. doi: 10.2196/90823 (PMC13249062; doi:10.2196/90823)
Supplement: Multimedia Appendix 2 [file periop-v9-e90823-s002.docx]

**Appendix 3:** VISION Post-operative complications and baseline variable definitions

MINS was defined as any myocardial infarction (as defined below), and any elevated troponin (higher than the local lab threshold) judged to be due to myocardial ischemia (i.e. without evidence of a non-ischemic etiology [e.g. chronic elevation, pulmonary embolism, sepsis, cardioversion]) that occurred within the first 30 days after the initiation of surgery. The only exceptions to the definition of an elevated troponin will be to use a higher threshold for troponin T (TnT) of ≥30 ng/L, and for high-sensitivity troponin T (hsTnT) of 20 to <65 ng/L with an absolute change of at least 5 ng/L or an hsTnT level ≥65 ng/L.  These threshold for TnT and hsTnT are based upon data from a large international prospective perioperative cohort study that established troponin thresholds that were independently associated with 30-day mortality after noncardiac surgery.

Myocardial Infarction was diagnosed if any one of the following criteria were met:

1. Detection of a rise or fall of a cardiac biomarker (preferably troponin) with at least one value above the 99th percentile of the upper reference limit (URL) together with evidence of myocardial ischemia with at least one of the following:
   - - - 1. ischemic signs or symptoms (i.e., chest, arm, neck, or jaw discomfort; shortness of breath, pulmonary edema);
         2. development of pathologic Q waves present in any two contiguous leads that are ≥ 30 milliseconds;
         3. new or presumed ECG changes indicative of ischemia (i.e., ST segment elevation [≥ 2 mm in leads V1, V2, or V3 OR ≥ 1 mm in the other leads], ST segment depression [≥ 1 mm], or symmetric inversion of T waves ≥ 1 mm) in at least two contiguous leads;
         4. new left bundle branch block (LBBB); or
         5. new cardiac wall motion abnormality on echocardiography or new fixed defect on radionuclide imaging
         6. identification of intracoronary thrombus on angiography or autopsy
2. Cardiac death, with symptoms suggestive of myocardial ischemia and presumed new ischemic ECG changes or new LBBB, but death occurred before cardiac biomarkers were obtained, or before cardiac biomarker values would be increased.
3. Percutaneous coronary intervention (PCI) related myocardial infarction is defined by elevation of a troponin value (>5 x 99th percentile URL) in patients with a normal baseline troponin value (≤99th percentile URL) or a rise of a troponin measurement >20% if the baseline values are elevated and are stable or falling. In addition, either (i) symptoms suggestive of myocardial ischemia or (ii) new ischemic ECG changes or (iii) angiographic findings consistent with a procedural complication or (iv) imaging demonstration of new loss of viable myocardium or new regional wall motion abnormality are required.

1. Stent thrombosis associated with myocardial infarction when detected by coronary angiography or autopsy in the setting of myocardial ischemia and with a rise and/or fall of cardiac biomarker values with at least one of value above the 99th percentile URL.
2. Coronary artery bypass grafting (CABG) related myocardial infarction is defined by elevation of cardiac biomarker values (>10 x 99th percentile URL) in patients with a normal baseline troponin value (≤99th percentile URL). In addition, either (i) new pathological Q waves or new LBBB, or (ii) angiographic documented new graft or new native coronary artery occlusion, or (iii) imaging evidence of new loss of viable myocardium or new regional wall motion abnormality.
3. For patients who are believed to have suffered a myocardial infarction within 28 days of a MINS event or within 28 days of a prior myocardial infarction, the following criterion for myocardial infarction is required:

Detection of a rise or fall of a cardiac biomarker (preferably troponin) with at least one value above the 99th percentile of the upper reference limit (URL) and 20% higher than the last troponin measurement related to the preceding event together with evidence of myocardial ischemia with at least one of the following:

- - - - 1. ischemic signs or symptoms (i.e., chest, arm, neck, or jaw discomfort; shortness of breath, pulmonary edema);
        2. development of pathologic Q waves present in any two contiguous leads that are > 30 milliseconds;
        3. new or presumed new ECG changes indicative of ischemia (i.e., ST segment elevation [> 2 mm in leads V1, V2, or V3 OR > 1 mm in the other leads], ST segment depression [> 1 mm], or symmetric inversion of T waves > 1 mm) in at least two contiguous leads;
        4. new LBBB; or
        5. new cardiac wall motion abnormality on echocardiography or new fixed defect on radionuclide imaging
        6. identification of intracoronary thrombus on angiography or autopsy

Venous thromboemboli (VTE) was diagnosed if the patient had either a pulmonary embolism or deep vein thrombosis as defined below:

Pulmonary embolism (PE) was diagnosed if any of the following were true:

1. A high probability ventilation/perfusion lung scan

2. An intraluminal filling defect of segmental or larger artery on a helical CT scan

3. An intraluminal filling defect on pulmonary angiography

4. A positive diagnostic test for DVT(i.e., positive compression ultrasound) and a non-diagnostic (i.e.,low or intermediate probability) ventilation/perfusion lung scan

5. A positive diagnostic test for DVT(i.e., positive compression ultrasound) and a non-diagnostic (i.e., subsegmental defects or technically inadequate study) helical CT scan

Deep vein thrombosis (DVT) was diagnosed if any of the following were true:

1. A persistent intraluminal filling defect on contrast venography

2. Non-compressibility of one or more venous segments on B mode compression ultrasonography

3. A clearly defined intraluminal filling defect on contrast enhanced computed tomography

Stroke was diagnosed in patients who developed a new focal neurological deficit thought to be vascular in origin with signs and symptoms lasting more than 24 hours.

Acute kidney injury (AKI) resulting in dialysis was diagnosed if there were any new acute renal failure requiring the use of dialysis within 30-days of major general surgery. Dialysis was defined as the use of a hemodialysis machine or peritoneal dialysis apparatus.

Infection was defined as a pathologic process caused by the invasion of normally sterile tissue or fluid or body cavity by pathogenic or potentially pathogenic organisms.

Sepsis was defined as the presence of infection and a systemic inflammatory response. Systemic inflammatory response requires 2 or more of the following factors: core temperature > 38 ºC or < 36 ºC; heart rate > 90 bpm; respiratory rate > 20 breaths/min; white blood cell count > 12 x 10^9^/ L or < 4 x 10^9^/ L.

Atrial fibrillation was diagnosed when a patient experienced a new AF episode within 30-days of general surgery that resulted in angina, congestive heart failure, symptomatic hypotension, or that required treatment with a rate controlling drug, antiarrhythmic drug, or electrical cardioversion.

Congestive heart failure (CHF) was diagnosed if a patient developed at least one of the following clinical signs: elevated jugular venous pressure, respiratory rales/crackles, crepitations, or presence of S3 AND at least one of the following radiographic findings: vascular redistribution, interstitial pulmonary edema, or frank alveolar pulmonary edema.

Bleeding was defined as bleeding which results in postoperative hemoglobin <70g/L, or leads to a transfusion of at least one unit of packed red blood cell (pRBC), reoperation, or is thought to be the cause of death.

Recent high risk coronary artery disease – Diagnosis ≤ 6 months prior to noncardiac surgery of: a myocardial infarction, acute coronary syndrome, Canadian Cardiovascular Society Class (CCSC) III angina or CCSC IV angina.

CCSC III angina – angina occurring with level walking of 1-2 blocks or climbing ≤ 1 flight of stairs at a normal pace

CCSC IV – inability to perform any physical activity without the development of angina

COPD – Noted if the chart or a physician had ever indicated that a patient has had chronic bronchitis. If there is no mention of this but the patient states they have had daily production of sputum for at least 3 months in 2 consecutive years then they were marked as having COPD. If a physician has ever indicated that a patient has emphysema or if a patient's Pulmonary Function Tests (PFT) stated fixed or irreversible airflow limitation and/or emphysema then they were marked as having COPD.

Peripheral vascular disease – A current or prior history of: physician diagnosed intermittent claudication, vascular surgery for atherosclerotic disease, an ankle/arm systolic blood pressure ratio ≤ 0.90 in either leg at rest, or angiographic or doppler study demonstrating ≥ 70%

stenosis in a noncardiac artery.

Active Cancer – Defined as a patient with a diagnosis of cancer who is or has received active treatment for their cancer (e.g., chemo, radiation, or surgery ) within the previous 6 months; however, it does not apply to patients with non-melanoma skin cancers. Examples of surgery to treat active cancer include resection of primary or metastatic tumour, palliative surgery such as intestinal bypass to relieve symptoms, or reconstructive surgery. It does not apply to surgery for a biopsy.
